# Multi-Mycotoxin Analyses by UPLC-MS/MS in Wheat: The Situation in Belgium in 2023 and 2024

**DOI:** 10.3390/foods14132300

**Published:** 2025-06-28

**Authors:** Camille Jonard, Anne Chandelier, Damien Eylenbosch, Joke Pannecoucque, Bruno Godin, Caroline Douny, Marie-Louise Scippo, Sébastien Gofflot

**Affiliations:** 1 Walloon Agricultural Research Centre, 5030 Gembloux, Belgiuma.chandelier@cra.wallonie.be (A.C.); d.eylenbosch@cra.wallonie.be (D.E.); b.godin@cra.wallonie.be (B.G.); s.gofflot@cra.wallonie.be (S.G.); 2 Research Institute for Agriculture, Fisheries and Food, 9820 Merelbeke-Melle, Belgium; joke.pannecoucque@ilvo.vlaanderen.be; 3 Laboratory of Food Analysis, Fundamental and Applied Research for Animal & Health, Veterinary Public Health, University of Liège, 4000 Liège, Belgium; mlscippo@uliege.be

**Keywords:** mycotoxins, UPLC-MS/MS, winter wheat, Belgium

## Abstract

This work proposes an insight into the mycotoxins detected in wheat from the 2023 and 2024 harvests in Belgium and highlights the link between agronomic conditions and mycotoxin contamination. The study utilized samples from a Belgian trial network, covering nine locations in 2023 and eight in 2024, ensuring diverse pedoclimatic contexts and including 11 different varieties. Sowing and harvest dates, previous crops and meteorological data were collected for these locations. A validated UPLC-MS/MS multi-mycotoxin method able to detect 20 mycotoxins, regulated or not, was used. Deoxynivalenol, zearalenone, and enniatins B and B1 were detected in the 2023 and 2024 samples. Enniatin A1 was only detected in the 2024 samples. Mycotoxin contamination was higher in 2024 compared to 2023, in terms of both the number of contaminated samples and the contamination levels. Enniatins B and B1, non-regulated mycotoxins, were widely detected in the 2024 wheat samples, with enniatin B detected in 68 out 88 samples ranging from 12 to 488 µg/kg. Differences between the wheat varieties were observed, with some varieties showing significantly higher contamination. Additionally, geographic location appeared to influence contamination levels, which could be related to previous crops or meteorological events. In conclusion, this research provides a comprehensive analysis of mycotoxin co-contamination in wheat samples from diverse pedoclimatic contexts in Belgium based over 2 years. It shows the importance of weather conditions on mycotoxin contamination. It also emphasizes the importance of variety selection to manage mycotoxin contamination.

## 1. Introduction

Mycotoxins are secondary metabolites produced by fungal genera such as *Aspergillus* spp., *Alternaria* spp., *Fusarium* ssp. and *Penicillium* spp. [1,2]. More than 300 mycotoxins have been reported in the literature. Among mycotoxins, the most studied compounds are aflatoxins (AFs), deoxynivalenol (DON), ochratoxin A (OTA) and zearalenone (ZEA), because of their toxicity to both animals and humans, their occurrence and because they are regulated [3,4]. In addition, some emerging mycotoxins, such as enniatins (ENNs), beauvericin (BEA) or sterygmatocystin (STE), defined as not regulated or not routinely monitored mycotoxins, are currently more frequently observed in raw agricultural products, used as food or feed [5,6].

DON, also called vomitoxin, is mainly produced by *Fusarium graminearum*. The main *Fusarium* species that produce ZEA are *F. graminearum*, *F. crookwellense*, *F. sporotrichioides* and *F. culmorum*. DON and ZEA can co-occur as they are produced by the same *Fusarium* species [7].

Ochratoxins A and B (OTB) are produced by species of genera *Aspergillus* and *Penicillium* [8]. The main producers of AFs are *Aspergillus flavus* and *Aspergillus parasiticus*. Among the AFs, the most widespread and the most dangerous molecule is aflatoxin B1 (AFB1) [9,10].

ENNs are produced by different *Fusarium* species such as *F. avenaceum*, *F. oxysporum*, *F. poae* or *F. tricinctum* [11]. Currently, ENNs are not regulated. The last European Food Safety Authority (EFSA) report concluded that there was a lack of acute toxicity and genotoxicity data [12].

Mycotoxins can be found in various foodstuffs such as coffee, beans, nuts, spices, maize, wheat, etc. [13]. Mycotoxin contamination is a worldwide issue for human and animal health, crop cultivation and meat production [10,14]. Mycotoxins are thermally stable, meaning that they are not destroyed during food processing [15,16,17]. Consumption of contaminated products may lead to bioaccumulation of the toxins in the organs, leading to acute or chronic effects on humans and animals depending on the dose and duration of exposure [10,15,18]. The possible effects can be mutagenic, teratogenic, carcinogenic, hepatotoxic, cytotoxic or immunosuppressive [6,7,8]. Therefore, some mycotoxin concentrations are regulated worldwide and controlled in food [2]. In the European Union, the Commission Regulation 2023/915 [19] and its amendments [20,21] define the maximum levels (MLs) of mycotoxins in food. For unprocessed cereals, the ML for AFB1 is set at 2 µg/kg, while the total concentration of AFs must not exceed 4 µg/kg. The combined concentration of fumonisins B1 and B2 (FUM) is limited to 1000 µg/kg. Additionally, the MLs are 5 µg/kg for OTA, 1000 µg/kg for DON, 100 µg/kg for ZEA and 50 µg/kg for the sum of T-2 and HT-2 toxins. In feed, Directive 2002/32/EC of the European Parliament and its amendments define maximum contents for certain mycotoxins [22]. The MLs are 250 µg/kg for OTA, 8000 µg/kg for DON, and 2000 µg/kg for ZEA. These regulations are updated in line with advances in knowledge about mycotoxins’ toxic effects.

Mycotoxin contamination can occur during pre-harvest, harvest and storage stages if the conditions are appropriate [13,23]. Infections by toxigenic fungi are influenced by weather conditions, especially humidity and temperature, agricultural practices, insect damage and storage conditions [5,23,24]. In cereals, heavy rains, leading to high air humidity, during flowering can induce *Fusarium* infection of ears, which can lead to higher mycotoxin content in the plants [25]. *Fusarium* growth increases at temperatures between 20 °C and 30 °C and in a matrix with water activity above 0.88 [25]. The probability of contamination with ZEA increases with heavy precipitation and long cool periods [23]. In addition, late harvest coupled with favorable weather conditions may enhance ZEA production [26]. On the other hand, *Aspergillus* growth is enhanced under warm temperatures, between 25 °C and 35 °C, and dry weather [27]. *Aspergillus* mycotoxin and FUM productions are higher above 22 °C [27]. Dry weather and late season rains may lead to FUM production [25].

Wheat is one of the most widely cultivated crops in the world [28]. It is a major source of carbohydrates and essential micro-nutrients in the human diet [28,29]. In 2023, the Food and Agriculture Organization (FAO) estimated the global cereal production at 2847 million tons [30]. In Europe, an average of 26 million hectares of wheat is cultivated, leading to the production of approximatively 254.6 million tons for food and feed ingredients [30]. The worldwide average amount of wheat consumption per capita is 65.6 kg annually, and it is the second most consumed cereal after rice [28]. In Europe, 43.8% of the wheat produced is used for food and 36% for feed, while the other 20.2% is used for seed, processing and other uses, or subjected to losses [28]. In Wallonia, the southern part of Belgium, the situation is different, as only 10% of the wheat is used for food, 45% for feed and 45% for fuel [31].

The most prevalent mycotoxins in wheat are DON, ZEA, AFB1, OTA, HT2, T2, AFs and FUMs [24]. In addition, some emerging mycotoxins have also been detected in wheat, such as ENNs, BEA and moniliformin [7]. Co-occurrence of mycotoxins is a common phenomenon, as fungal species can produce multiple mycotoxins [3].

Mycotoxins are one of the most important issues regarding food safety, as they are observed worldwide in crops and can cause adverse effects on human health [32]. In addition, fungal infection and mycotoxin contamination have a huge economic impact on agriculture and the food industry, as they can lead to a decrease in production yield or destruction of contaminated products [23,24,33]. Up to 60 to 80% of food crops are contaminated with mycotoxins, depending on the mycotoxin [34]. Furthermore, due to climate change, mycotoxin contamination patterns tend to evolve, and mycotoxin contamination is expected to increase [24,35,36]. Therefore, continuous monitoring and analysis of conventional and emerging mycotoxins is of paramount importance.

Different methods allow mycotoxin detection in food matrices. Many chromatographic technics can be used for this purpose [37]. More specifically, liquid chromatography (LC) coupled with tandem mass spectrometry (UPLC-MS/MS) is one of the most widely used analytical methods for multi-targeted mycotoxin monitoring, thanks to its accuracy, even in complex matrices [38]. Modern chromatographic technics can also be used to analyze mycotoxins such as nano-LC to increase sensibility and decrease sample volume. However, such technics require a higher clean-up of the sample to avoid system clogging [37]. For untargeted analysis, high-resolution liquid chromatography is generally used.

Currently, mycotoxin contamination patterns in Belgium remain poorly understood. As these patterns are likely to evolve with climate change, it is important to investigate the current situation. This study provides an overview of the occurrence of mycotoxins in wheat harvested in Belgium in 2023 and 2024, two agronomic years characterized by markedly different rainfall patterns during anthesis and harvest, which are critical periods for fungal contamination and mycotoxin production. Various wheat samples were collected at diverse locations and analyzed using a validated multi-mycotoxin analysis method by UPLC-MS/MS.

## 2. Materials and Methods

### 2.1. Chemicals and Reagents

All chemicals and reagents used in this study were of analytical grade: Milli-Q water (Milli-Q^®^ IQ 7010 purification system) (MilliporeSigma, Burlington, MA, USA), acetonitrile AR (Biosolve, Dieuze, France), acetonitrile MS/MS grade (Biosolve, Dieuze, France), acetic acid AR (Fisher Chemical, Merelbeke, Belgium), methanol MS/MS grade (Biosolve, Dieuze, France), formic acid (Avantor, VWR, Leuven, Belgium), ammonium acetate (Fluka, Mint Street, NC, USA), MgSO_4_ (Roth, Karlsruhe, Germany) and NaCl (Roth, Karlsruhe, Germany).

Mycotoxin standard solutions of known concentration for 15-acetyldeoxynivalenol (15Ac-DON), 3-acetyldeoxynivalenol (3Ac-DON), DON, HT2, alternariol (AOH), alternariol-monomethyl-ether (AME), T2, ZEA, OTA, OTB, FUM B1 and STE were purchased from Romer-Labs (Romer-Labs, Getzersdorf, Austria). ENNs (A, A1, B and B1) standards were purchased from Merck (Merck KGaA, Darmstadt, Germany).

### 2.2. Sample Collection

A total of 45 samples of wheat grains from the 2023 harvest and 88 samples from the 2024 harvest were analyzed.

In 2023, the samples came from a trial network having nine different locations distributed across Belgian territory (Thorembais, Hannut, Gesves, Terwagne, Enghien, Leffinge, Poperinge, Merelbeke and Bassevelde). This trial network was set up by CRA-W and ILVO for variety registration in Belgium. These trials aim to compare varieties in contrasting pedoclimatic growth conditions and are conducted without fungicide protection or growth regulator. Varieties are cultivated in microplots (11 to 15 m^2^, depending on the trials) and are harvested using a special combine harvester, which allows the separate harvesting of each plot and avoids mixing the samples taken from each plot. From each location, five varieties were analyzed.

**Figure 1:**
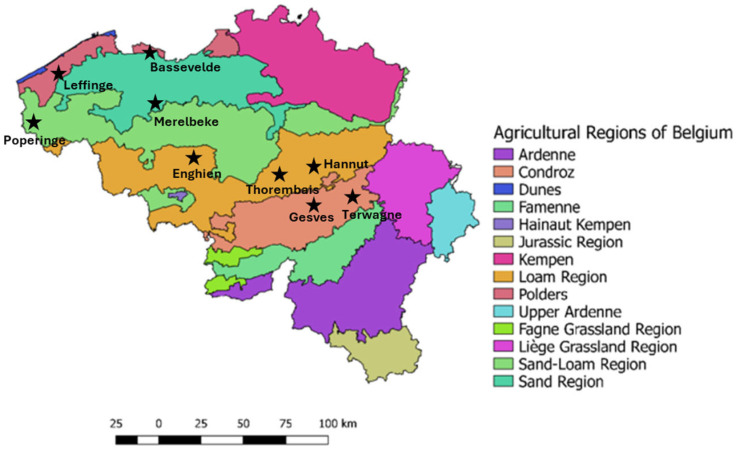
Pedologic map of Belgium with the location of the cultivation sites. Adapted from reference [39].

In 2024, the samples were collected from the same trial network from only eight different locations, as the Bassevelde field could not be sown due to poor climatic conditions in winter 2023. For each location, 11 varieties were collected. The different locations of sample collection are presented in Figure 1.

The trials were sown between October and December and the samples were harvested in July and August, depending on the location. The sowing and harvest dates, the presence or absence of ploughing, and previous crops cultivated on the field are presented in Table 1.

Flowering and maturity dates have been estimated based on the Phénoblé tool (CRA-W, Gembloux, Belgium). In 2023, wheat flowering occurred between the 7th and the 10th of June, and the wheat reached maturity around the 20th of July. In 2024, flowering occurred between the 2nd and the 4th of June, and maturity was reached around the 22nd of July.

### 2.3. Meteorological Considerations

During the first season of sampling, October and November 2022 were dry and warm in Belgium, while December was marked by several days of frost. Precipitation remained within normal levels. On average, January 2023 was mild and wet, whereas February was particularly dry. The spring of 2023 was extremely rainy, with a colder-than-average April. From mid-May to the end of June, at the time of ear emergence and flowering of wheat crops, the weather was very dry and sunny. Late July and early August had significant rainfall, delaying much of the harvest.

During the second season of sampling, rain returned from mid-October 2023 onward, delaying most sowing activities. The winter was very wet and generally mild, except for a period of frost and snow in mid-January. Spring of 2024 was extremely rainy, as was June. However, precipitation decreased in July and August, allowing for a successful wheat harvest under good conditions.

**Figure 2:**
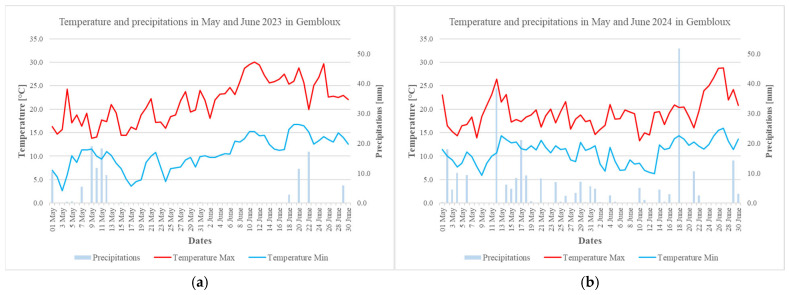
Temperature (°C) and precipitation (mm) in May and June 2023 (**a**) and 2024 (**b**) in Gembloux. Data from IRM station in Gembloux.

Daily temperatures and precipitations in May and June 2023 and 2024 are presented in Figure 2.

### 2.4. Sample Preparation

The mycotoxin analysis was performed on whole-wheat flour, obtained after milling approximately 50 g of wheat grains using a laboratory mill (Cyclotec, FOSS, Nanterre, France). The resulting flour had a particle size under 1 mm.

The extraction procedure was adapted from the QuEChERS method [40,41,42,43,44].

Five grams of whole-wheat flour was weighed in Falcon tubes (50 mL) and 10 mL of water was added. After a one-hour rest, 10 mL of acetonitrile solution containing 1% acetic acid was introduced in each tube. The samples were then agitated using a Mixer Mill MM400 (Retsch GmbH, Aartselaar, Belgium) for 10 min at 10 Hz. Thereafter, 4 g of MgSO_4_ and 1 g of NaCl were added to the tubes. The tubes were hand shaken and then agitated using a Mixer Mill MM400 for 2 min at 10 Hz. After stirring, the samples were centrifuged for 5 min at 3000 g (Centrifuge 5810 R, Eppendorf, Leipzig, Germany).

A volume of 100 µL of the supernatants was collected and diluted with 900 µL of a 50:50 (*v*/*v*) solution of water and methanol into injection vials.

### 2.5. Standard Solutions and Calibration

Two mycotoxin pools were constituted by diluting the initial individual standard solution in acetonitrile. The first pool was composed of 15Ac-DON, 3Ac-DON, DON, HT2, AOH, AME, T2, ZEA, OTA and OTB, with 15Ac-DON, 3Ac-DON, DON, HT2, AOH, AME, T2, ZEA at 100 µg/kg, DON at 1000 µg/kg and OTA and OTB at 5 µg/kg. The second pool was prepared including the following emerging mycotoxins: ENNs (A, A1, B, B1), FUM B1 and STE, all at 100 µg/kg.

To address potential matrix effects, matrix-matched calibration curves were established by spiking mycotoxins into a sample extract free of the targeted mycotoxins.

Two different calibration curves were produced, one with each mycotoxin pool. The whole-wheat flour (used as a “blank” matrix) was extracted as described above. Specified volumes of blank matrix extract were introduced in 1.5 mL Eppendorf tubes. Then, specific volumes of the mycotoxin pool solution were added to the Eppendorf tubes to make a total volume of 1 mL. A volume of 100 µL of the obtained solution was sampled and 900 µL of a 50:50 (*v*/*v*) solution of water and methanol was added. Then, the solutions were transferred into injection vials. The mycotoxin concentrations used for the calibration curves are shown in Table 2.

### 2.6. UPLC-MS/MS Conditions

The UPLC-MS/MS conditions were optimized based on different studies such as [40,41,42,43].

The samples were injected into a Waters Acquity I-Class (Waters, Milford, MA, USA) UPLC system equipped with a BEH C18 column (2.1mm × 50 mm, 1.7 μm), coupled to a Xevo TQ-xS mass spectrometer (Waters, Milford, MA, USA). Mobile phase A was a 10 mM ammonium acetate–water solution containing 0.1% formic acid, and mobile phase B was methanol with 0.1% formic acid. The column temperature was set at 50 °C, the flow rate was 0.3 mL/min and the injection volume was 2 µL. The elution gradient started at 10% B, held for 3 min and increased linearly to 30% in 7 min; then increased again to 10% in 10 s and kept constant for 2 min. Finally, B decreased to 10% in 10 s and equilibrated for 3 min.

**Figure 3:**
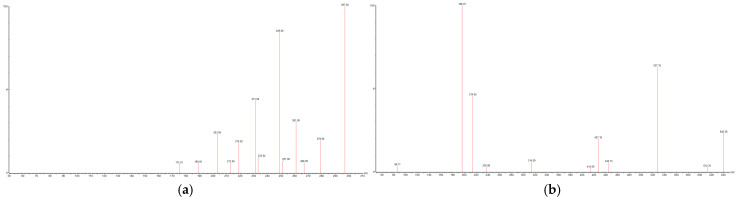
Mass spectra for DON (**a**) and ENN B (**b**).

The mass spectrometer was used in Multiple Reaction Monitoring (MRM) under positive ionization modes. Two transitions were selected for each mycotoxin, one for quantification and another for qualification. Regarding the mass spectrometer parameters, the capillary voltage was 3.4 kV, the desolvation temperature was 400 °C, the desolvation gas flow was 800 L/Hr, the cone gas flow was 150 L/Hr and the nebuliser pressure was 7 bar. The cone voltage and the collision energy were optimized for each transition by direct infusion. The transitions used for each mycotoxin and the associated energies and cone voltages are presented in Table 3. Examples of mass spectra for the DON and ENN B are shown in Figure 3.

### 2.7. Data Treatment

For each sample, the peaks from the obtained chromatograms were integrated using TargetLynx V4.2 (Waters, USA). The calibration curves were determined by plotting the peak area of the quantification transition versus the mycotoxin concentration and using the linear regression equation *y* = *ax* + *b*. An average calibration curve, calculated from the calibration curves injected before and after the samples (bracketed calibration), was used for quantification.

The sample concentrations were calculated based on the average calibration curve for each mycotoxin and corrected using the exact mass of the sample intake and the recovery of each mycotoxin determined during method validation.

Recoveries were determined by spiking samples with known amounts of each mycotoxin. Samples were spiked to obtain three levels of concentration according to the MLs for the regulated mycotoxins, ML/2, ML and 1.5× ML. For non-regulated mycotoxins, the central value was 100 µg/kg. The spiked samples were then extracted as described above. For each level of concentration and for each individual sample, the recovery, expressed as a percentage, was calculated as the ratio between the calculated concentration and theoretical concentration. These recoveries are presented in Table 4. The lowest points of calibration used as the limit of quantification (LOQ) and the limit of detection (LOD) determined during method validation are also presented in Table 4. For OTA and OTB, the second-lowest point of calibration was used as the LOQ, because signal-to-noise (S/N) ratio was too low for the lowest point of calibration. The LOD was determined by extrapolating a concentration corresponding to a S/N of 3, then experimentally verified by spiking standard solutions in blank matrices to adjusted concentrations until this ratio was achieved.

Each sample was extracted and analyzed twice. The difference between the two calculated concentrations was required not to exceed 20%. Otherwise, the sample was re-extracted in duplicate.

All the statistical analyses were conducted using RStudio, version 2024.12.0 (Posit software PBC, Boston, MA, USA). The differences between the wheat varieties and the wheat cultivation sites were assessed through a Kruskal–Wallis test followed by a Dunn test.

## 3. Results

### 3.1. Mycotoxin Occurrence in Samples Collected in 2023 and 2024

A total of 133 samples were analyzed using the UPLC-MS/MS multi-mycotoxins analytical method. In 2023 (Table 5), nine out of 45 samples, corresponding to one-fifth of the total, were contaminated with mycotoxins at concentrations exceeding the LOQ. The mycotoxins detected were DON, ZEA and ENN B; the other mycotoxins included in the method were not detected in these samples. Only two samples showed co-contamination by at least two mycotoxins.

In 2024 (Table 6), 74 out of 88 samples displayed higher concentrations than the LOQ for at least one mycotoxin, meaning that 84% of the samples contained quantifiable amounts of mycotoxins. DON, ZEA, ENN A1, ENN B and ENN B1 were the mycotoxins detected by UPLC-MS/MS. Co-contamination occurred in 59 samples. ENN B was the most prevalent mycotoxin in the 2024 samples. It was detected in 68 samples with concentrations ranging from 12 to 488 µg/kg. DON was detected in 52 samples, ENN B1 in 49, ENN A1 in 17 and ZEA in 10 samples. One sample was contaminated with DON at 1479 µg/kg, exceeding the level of 1000 µg/kg fixed by the European regulation for grains intended to be used for food [20]. No sample was over the limit for ZEA as the maximum level detected was 55 µg/kg, while the ML set in the European legislation is 100 µg/kg [19]. The maximum levels detected for ENN A1, B and B1 were 49, 488 and 185 µg/kg, respectively. Currently, ENNs are not regulated in Europe. Mycotoxin contamination was higher in 2024 than in 2023, in terms of both the number of contaminated samples and the contamination levels.

As few samples from the 2023 harvest were positive or had mycotoxin concentrations exceeding the LOQ, no further data analysis was performed. In 2024, most of the samples were contaminated by at least one mycotoxin, allowing for an assessment of the differences in contamination between varieties and cultivation sites. All the data are in the Supplementary Materials (Tables S1 and S2).

### 3.2. Differences in Concentration Between the Varieties in 2024 Wheat Samples

**Figure 4:**
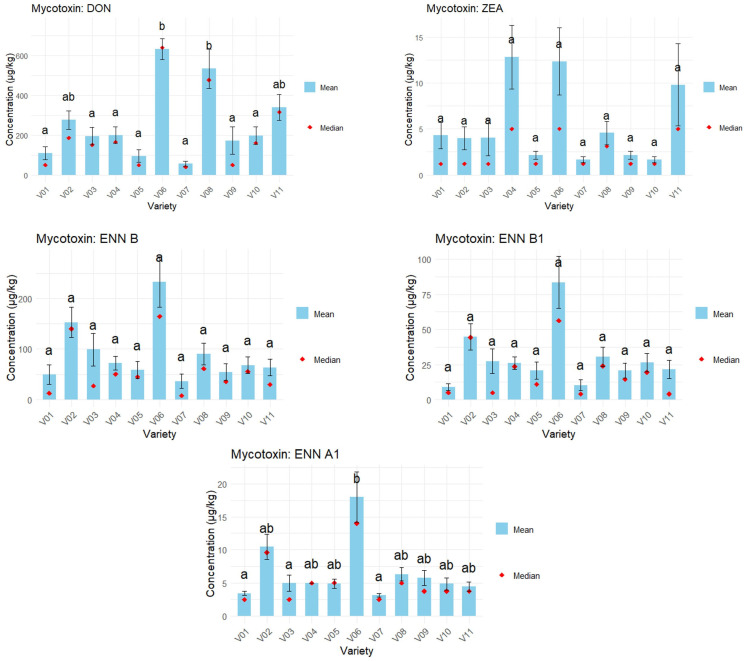
Mycotoxin contamination depending on the variety (n = 8 per variety) for 2024 wheat samples. Significance letters were assigned using a Dunn test performed after a Kruskal–Wallis test. Varieties sharing the same letter (a, ab or b) are not significantly different (*p* > 0.05). For the calculation of the mean, the middle-bound approach was used, meaning that for samples where the mycotoxin was not detected, the values were LOD/2 and for samples < LOQ, the values were LOQ/2.

For each mycotoxin, the differences in mycotoxin concentration between the varieties were assessed, as 11 different varieties of wheat were cultivated in each location. Hence, eight samples of each variety were available to be analyzed by UPLC-MS/MS. The results are shown in Figure 4 and the Supplementary Material (Table S2). The significance of the differences between the varieties was evaluated by performing a Kruskal–Wallis test (Table 7).

For ENN B, there was no significant difference in concentration between the varieties (*p* < 0.05). For the other mycotoxins (DON, ZEA, ENN B1 and ENN A1), a significant difference between the varieties was found.

Statistically, the DON concentration was higher in varieties 06 and 08. In addition, variety 06 was more contaminated with ENN A1 than the other varieties. For ENN B, ENN B1 and ZEA, the mycotoxin concentrations in the different varieties could not be statistically differentiated. However, variety 06 had the higher means among the analyzed varieties for ENN B and ENN B1. Therefore, within the scope of this work, considering the specific conditions of the 2024 study, variety 06 appears to be at a higher risk for mycotoxin contamination. On the other hand, variety 07 is among the least contaminated varieties for each mycotoxin; this variety seems to be more resistant to mycotoxin contamination. In addition, field observations are consistent with these results, as almost no wheat ears showed symptoms from *Fusarium* infection. However, these results should be confirmed with additional trials during subsequent years.

### 3.3. Differences in Mycotoxin Concentration Between the Cultivation Sites in 2024 Wheat Samples

**Figure 5:**
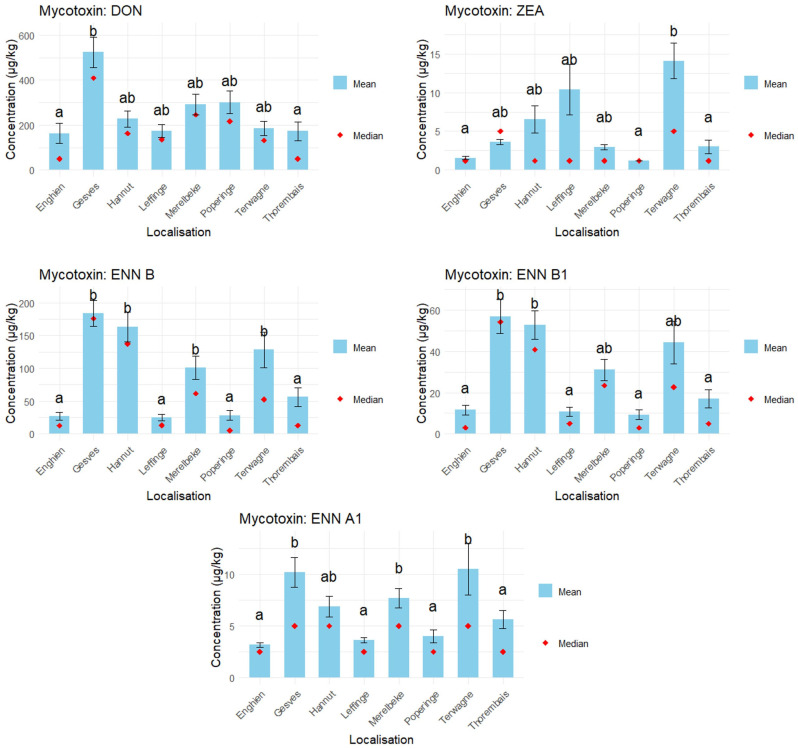
Mycotoxin contamination depending on the location for 2024 wheat samples (n = 11 per location). Significance letters were assigned using a Dunn test performed after a Kruskal–Wallis test. Varieties sharing the same letter (a, ab or b) are not significantly different (*p* > 0.05). For the calculation of the mean, the middle-bound approach was used, meaning that for samples where the mycotoxin was not detected, the values were LOD/2 and for samples < LOQ, the values were LOQ/2.

The location effect was studied by comparing the contamination level for each mycotoxin among the 11 varieties harvested in each location (Figure 5). The significance of the differences between the locations was evaluated by a Kruskal–Wallis test (Table 8).

For each mycotoxin, the cultivation site had a significant effect on the concentration. Within the scope of this work, for DON contamination, the most critical cultivation site was Gesves, and for ZEA it was Terwagne. For ENNs, mycotoxin contamination was significantly higher in Gesves, Hannut, Terwagne and Merelbeke. In 2024, Gesves seemed to be the most affected growing site for all mycotoxins. On the other hand, for ENNs, Enghien, Leffinge, Poperinge and Thorembais were less affected by mycotoxin contamination. For DON and ZEA, the sites the least affected by mycotoxin contamination were Enghien and Thorembais. Poperinge was not subjected to ZEA contamination at all.

## 4. Discussion

### 4.1. Effect of the Year of Harvest of Wheat on the Mycotoxin Contamination

Samples collected from the 2024 harvest were more contaminated by mycotoxins in terms of number of contaminated samples and level of contamination. However, only one sample was contaminated at a level exceeding the European regulation for food. As reported in the literature [25,27,45], rainy conditions and high relative humidity during the flowering of the wheat may enhance fungal contamination and mycotoxin production. Hence, the higher contamination of the 2024 samples (approximatively 86%) compared to those from 2023 (20% of positive samples) may be explained by weather conditions, as the period from mid-May to June 2023 was dry in Belgium, whereas the spring of 2024 was rainy. However, European data on mycotoxin contamination in cereals are available and indicate no significant difference in contamination levels between 2023 and 2024. For example, for DON, Cargill reported that 88% of samples were contaminated in both 2023 and 2024, while dsm-Firmenich reported 77% in 2023 and 76% in 2024 [46,47]. In contrast, the present study, conducted within a more geographically restricted area, reveals greater interannual variation, likely due to the stronger influence of local weather conditions on fungal development and mycotoxin production.

Other factors may explain the difference in mycotoxin contamination of wheat between these two years. Climate change is often cited as a factor influencing mycotoxin contamination patterns, as shifts in temperature and seasonal variations can alter the wheat growth cycle, potentially making it more susceptible to contamination [48,49,50]. However, to have a better understanding of the impact of climate changes on mycotoxin contamination of wheat in Belgium, this study should be continued for several years.

Both in 2023 and 2024, the same mycotoxins were detected except for ENN A1, which was detected only in 2024. DON and ZEA are commonly present in wheat [45], and this study confirms this observation. ENNs were the most prevalent mycotoxins in the samples harvested in 2024, whereas they were only detected in seven samples in 2023. ENNs are considered emerging mycotoxins as they are not regulated or routinely analyzed. However, there is a growing concern about these mycotoxins as they are more often detected and at a higher concentration [50]. dsm-Firmenich highlights a notable global presence of ENN B, supporting the observation made in this study of increased ENN B levels in Belgian wheat samples from 2024 [47]. The EFSA report from 2014 indicates that acute exposure to ENNs is not a concern for human health [12]. However, the data are not sufficient to draw firm conclusions about the chronic exposure for these mycotoxins. Therefore, it is relevant to gather contamination data if a new risk assessment is performed.

### 4.2. Differences in Mycotoxin Concentration Between the Wheat Varieties

Significant differences in concentration for DON, ZEA, ENN A1 and ENN B1 were observed between the wheat varieties (*p* < 0.05). For each mycotoxin, the differences in mycotoxin concentration between the varieties were assessed, as 11 different varieties of wheat were cultivated in each location. Hence, eight samples of each variety were available to be analyzed by UPLC-MS/MS. The results are shown in Figure 4 and the Supplementary Material (Table S2). The significance of the differences between the varieties was evaluated by performing a Kruskal–Wallis test (Table 7). This highlights that certain varieties may be more susceptible to mycotoxin contamination than others. For example, studies showed that lower anther retention is linked with high resistance to fungal infection [51]. These trials should be repeated over several years to confirm that the observations are due to varietal effect and not only an annual effect. Such differences in susceptibility could be attributed to genetic factors influencing fungal infection and toxin production. These findings emphasize the importance of selecting and breeding varieties with enhanced fungal resistance, which is one of the criteria for the acceptance and commercialization of new varieties. Incorporating resistance traits into breeding programs contributes to reducing mycotoxin contamination and improving the overall safety and quality of harvested grains. The selection of mycotoxin-resistant varieties represents a more effective strategy than fungicide application for reducing the risk of contamination.

### 4.3. Differences in Mycotoxin Concentration Between the Wheat Cultivation Sites

There is a significant effect of location on the mycotoxin presence in wheat. The location effect was studied by comparing the contamination level for each mycotoxin among the 11 varieties harvested in each location (Figure 5). The significance of the differences between the locations was evaluated by a Kruskal–Wallis test (Table 8).

The most impacted site in 2024 was Gesves, which had been subjected to heavy rainfall and mudflows that had damaged the cultivation site. Other significantly affected sites included Hannut, Terwagne and Merelbeke. Each site belongs to a different pedological region, except for Gesves and Terwagne, which are situated in Condroz. From these data, no clear relationship can be established between the pedological region and mycotoxin contamination. Based on crop history data from the past 7 years, the Terwagne site has frequently been cultivated with wheat and silage maize. This cropping pattern may have contributed to the buildup of a local inoculum. In contrast, silage maize was not present at the other Walloon sites.

At this stage, no obvious link can be established with the previous crop, sowing, ploughing or harvest dates. It could be interesting to investigate whether the same sites are the most affected by fungal contamination and mycotoxin presence each year, while others appear to be better preserved. Therefore, more data should be collected in the coming years to draw broader conclusions about the link between mycotoxin contamination and agricultural factors, helping to mitigate specific effects.

## 5. Conclusions

This study provides updated data on mycotoxin contamination in wheat harvested in Belgium during the 2023 and 2024 growing seasons. A total of 113 samples were analyzed, revealing a higher occurrence of mycotoxins in 2024, particularly ENNs. Notably, one sample exceeded the European ML for DON in 2024, while all the samples from 2023 remained below the regulatory thresholds. These findings underscore the strong influence of weather conditions, especially the rainy period during wheat flowering in 2024, which likely promoted fungal growth and subsequent mycotoxin production. Although climate change is frequently cited as a driver of such contamination patterns, long-term monitoring will be essential to better evaluate its specific impact in Belgium.

The results also highlight the importance of wheat variety selection and cultivation site conditions in managing mycotoxin risk. Certain varieties showed different levels of susceptibility to contamination, reinforcing the need for breeding programs focused on improving resistance to fungal pathogens. Additionally, annual variations in climate can significantly influence mycotoxin development, making some locations appear more or less favorable depending on specific weather events rather than inherent site characteristics.

By providing a two-year comparative dataset, this study provides an overview of mycotoxin occurrence in wheat in Belgium. Multiple factors may influence the mycotoxin presence in crops, such as weather (temperature and humidity), agricultural practices, previous crops, pests, etc., making it difficult to fully understand the underlying mechanisms. To better understand these trends and develop effective mitigation strategies, further data collection over multiple years is essential. This will provide a clearer picture of potential contamination patterns and help refine agricultural practices to minimize mycotoxin risks and ensure safer wheat production in the future.

## Figures and Tables

**Table 1:** Agricultural data for the trial network in 2023 (a) and 2024 (b).

(**a**)				
**Harvest 2023**				
	**Sowing Date**	**Harvest Date**	**Previous Crop**	**Ploughing**
Gesves	5 November 2022	10 August 2023	Potatoes	No
Terwagne	31 October 2022	14 August 2023	Sugar beet	No
Hannut	25 October 2022	13 August 2023	Flax	No
Thorembais	26 October 2022	11 August 2023	Flax	Yes
Enghien	16 October 2022	20 July 2023	Flax	No
Merelbeke	18 October 2022	18 July 2023	Potatoes	Yes
Poperinge	19 October 2022	18 July 2023	Corn silage	Yes
Leffinge	19 October 2022	26 July 2023	Potatoes	Yes
Bassevelde	19 October 2022	19 July 2023	Grain corn	Yes
(**b**)				
**Harvest 2024**				
	**Sowing Date**	**Harvest Date**	**Previous Crop**	**Ploughing**
Gesves	18 December 2023	12 August 2024	Potatoes	Yes
Terwagne	17 October 2023	06 August 2024	Rapeseed	No
Hannut	18 December 2023	06 August 2024	Sugar beet	No
Thorembais	22 November 2023	30 July 2024	Flax	Yes
Enghien	1 December 2023	30 July 2024	Canned peas	Yes
Merelbeke	18 October 2023	29 July 2024	Potatoes	Yes
Poperinge	18 December 2023	30 July 2024	Sugar beet	Yes
Leffinge	18 October 2023	30 July 2024	Potatoes	Yes

**Table 2:** Mycotoxin concentrations in µg/kg in the calibration curve.

	1 [µg/kg]	2 [µg/kg]	3 [µg/kg]
Standard 01	0.5	10	100
Standard 02	1.0	20	200
Standard 03	2.5	50	500
Standard 04	5.0	100	1000
Standard 05	7.5	150	1500
Standard 06	10	200	2000
Standard 07	15	300	3000

1 = ochratoxins (A and B); 2 = AME, AOH, 3Ac-DON, 15Ac-DON, T2, HT2, ZEA, ENN (A, A1, B, B1), FUM B1 and B2, citrinin, moniliformin, STE; 3 = DON.

**Table 3:** Optimized MS/MS parameters for the multi-mycotoxin detection method in MRM mode.

Mycotoxin	Molecular Mass	MODE	*m*/*z*	Cone (V)	Daughter 1	Energy1 (eV)	Daughter 2	Energy2 (eV)	RT (min)
AME	272.3	[M+H]^+^	273.0	20	128.0	60	228.0	40	10.9
AOH	258.2	[M+H]^+^	259.0	10	185.0	28	213.0	28	9.2
DON	296.3	[M+H]^+^	297.1	15	231.1	18	249.1	18	3.9
3Ac-DON	338.4	[M+H]^+^	339.1	25	137.0	15	203.1	12	7.0
15Ac-DON	338.4	[M+NH_4_]^+^	356.2	5	137.0	16	261.0	14	7.0
ZEA	318.4	[M+H]^+^	319.2	20	185.1	25	187.1	19	10.6
T2	466.5	[M+NH_4_]^+^	484.2	7	215.0	18	305.1	12	10.4
HT2	424.5	[M+NH_4_]^+^	442.2	5	215.0	12	263.1	10	9.8
OTA	403.8	[M+H]^+^	404.1	25	239.1	25	357.9	15	10.5
OTB	369.4	[M+H]^+^	370.0	10	187.0	30	204.8	20	9.7
Enniatin A	681.9	[M+H]^+^	682.6	30	99.6	40	209.8	25	11.8
Enniatin A1	667.9	[M+H]^+^	669.1	30	99.6	40	210.0	25	11.7
Enniatin B	639.8	[M+H]^+^	640.7	15	85.9	40	196.0	20	11.5
Enniatin B1	653.8	[M+H]^+^	655.2	20	85.9	40	196.1	25	11.6
Fumonisin B1	721.8	[M+H]^+^	722.3	15	334.3	35	352.3	35	10.0
Sterigmatocystin	324.28	[M+H]^+^	325.0	15	253.1	38	281.1	35	10.8

**Table 4:** Average recovery, limit of quantification (LOQ) and limit of detection (LOD) determined during method validation for the different mycotoxins.

Mycotoxins	Recovery [%]	LOQ [µg/kg]	LOD [µg/kg]
15Ac-DON	49	10	3.8
3Ac-DON	59	10	3.3
DON	88	100	60
AOH	90	10	8
AME	90	10	8
T2 + HT2	84	10	0.3
ZEA	96	10	2.4
OTA	104	1	0.5
OTB	99	1	0.5
ENN A	91	10	8
ENN A1	93	10	5
ENN B	93	10	1
ENN B1	92	10	6
FUM B1	58	10	3
STE	91	10	1.5

**Table 5:** Mycotoxin contamination of the 2023 wheat samples (n = 45).

Mycotoxin	ND (n)	<LOQ (n)	>LOQ (n)	Mean ± SD (µg/kg)	Min–Max of Samples > LOQ (µg/kg)	Median of Samples > LOQ (µg/kg)
DON	39	1	5	63 ± 104	171–567	317
ZEA	40	0	5	2.7 ± 4.4	12–18	15
ENN B	38	5	2	4.5 ± 19.7	28–131	79
ENN B1	44	1	0	3.0 ± 0.3	All < LOQ	All < LOQ

n: number of samples (n total = 45); ND: non-detected; for the calculation of the mean, the middle-bound approach was used, meaning that for ND samples, the values were LOD/2 and for samples < LOQ, the values were LOQ/2; the min, max and median included samples above the LOQ only.

**Table 6:** Mycotoxin contamination of the 2024 wheat samples (n = 88).

Mycotoxin	ND (n)	<LOQ (n)	>LOQ (n)	Mean ± SD (µg/kg)	Min–Max of Samples > LOQ (µg/kg)	Median of Samples > LOQ (µg/kg)
DON	11	25	52	255 ± 268	131–1478	370
ZEA	53	25	10	5.4 ± 9.5	16–55	28
ENN A1	35	36	17	6.5 ± 7.0	10–49	14
ENN B	3	17	68	89 ± 109	12–488	66
ENN B1	21	18	49	29 ± 37	7–185	38

n: number of samples (n total = 88); ND: non-detected; for the calculation of the mean, the middle-bound approach was used, meaning that for ND samples, the values were LOD/2 and for samples < LOQ, the values were LOQ/2; the min, max and median included samples above the LOQ only.

**Table 7:** Significance of the differences of mycotoxin contamination between the varieties in 2024 (Kruskal–Wallis test).

	Mean Concentration ± SD (µg/kg)	Kruskal–Wallis Χ^2^	df	*p*-Value	Significance
DON	255 ± 268	42.239	10	6.80 × 10^−6^	***
ZEA	5.4 ± 9.5	23.644	10	0.008604	**
ENN A1	6.5 ± 7.0	24.693	10	0.005958	**
ENN B	89 ± 109	16.969	10	0.07506	n.s.
ENN B1	29 ± 37	19.086	10	0.03918	*

df: degree of freedom. The differences between the varieties are considered significant (*) for *p*-value < 0.05, (**) for *p*-value < 0.01, (***) for *p*-value < 0.001, n.s. = non-significant.

**Table 8:** Significance of the differences of mycotoxin contamination of wheat between the cultivation sites in 2024 (Kruskal–Wallis test).

	Mean Concentration ± SD (µg/kg)	Kruskal–Wallis Χ^2^	df	*p*-Value	Significance
DON	255 ± 268	15.108	7	0.03464	*
ZEA	5.4 ± 9.5	29.384	7	0.0001231	***
ENN A1	6.5 ± 7.0	25.715	7	0.0005662	***
ENN B	89 ± 109	40.970	7	8.21 × 10^−7^	***
ENN B1	29 ± 37	34.123	7	1.63 × 10^−5^	***

df: degree of freedom. The differences between the varieties are considered significant (*) for *p*-value < 0.05, (***) for *p*-value < 0.001.

## Data Availability

The original contributions presented in this study are included in the article/Supplementary Material. Further inquiries can be directed to the corresponding author(s).

## Supplementary Materials

The following supporting information can be downloaded at https://www.mdpi.com/article/10.3390/foods14132300/s1, Table S1: Mycotoxin concentrations of 2023 wheat samples; Table S2: Mycotoxin concentration of 2024 wheat samples.

## Abbreviations

The following abbreviations are used in this manuscript:

15Ac-DON	15-acetyldeoxynivalenol
3Ac-DON	3-acetyldeoxynivalenol
AF	aflatoxin
AFB1	aflatoxin B1
AME	alternariol-monomethyl-ether
AOH	alternariol
BEA	beauvericin
df	degree of freedom
DON	deoxynivalenol
EFSA	European Food Safety Authority
ENN	enniatin
FAO	Food and Agriculture Organization
FUM	fumonisin
HT2	HT2 toxin
LC	liquid chromatography
LOD	limit of detection
LOQ	limit of quantification
ML	maximum level
MRM	Multiple Reaction Monitoring
ND	non-detected
OTA	ochratoxin A
OTB	ochratoxin B
S/N	signal-to-noise ratio
STE	sterygmatocystin
T2	T2 toxin
UPLC-MS/MS	Ultra Performance Liquid Chromatography coupled with tandem mass spectrometry
ZEA	zearalenone
